# Comparative SEM analysis of nine F22 aligner cleaning strategies

**DOI:** 10.1186/s40510-017-0178-9

**Published:** 2017-09-11

**Authors:** Luca Lombardo, Marco Martini, Francesca Cervinara, Giorgio Alfredo Spedicato, Teresa Oliverio, Giuseppe Siciliani

**Affiliations:** 1 Postgraduate School of Orthodontics, University of Ferrara, Via Montebello 31, Ferrara, 44100 Italy; 2 School of Orthodontics, University of Ferrara, Ferrara, Italy; 3 School of Economics, Management and Statistics, University of Bologna, Piazza Scaravilli 2, Bologna, 40121 Italy

## Abstract

**Background:**

The orthodontics industry has paid great attention to the aesthetics of orthodontic appliances, seeking to make them as invisible as possible. There are several advantages to clear aligner systems, including aesthetics, comfort, chairside time reduction, and the fact that they can be removed for meals and oral hygiene procedures.

**Methods:**

Five patients were each given a series of F22 aligners, each to be worn for 14 days and nights, with the exception of meal and brushing times. Patients were instructed to clean each aligner using a prescribed strategy, and sections of the used aligners were observed under SEM. One grey-scale SEM image was saved per aligner in JPEG format with an 8-bit colour depth, and a total of 45 measurements on the grey scale (“Value” variable) were made. This dataset was analysed statistically via repeated measures ANOVA to determine the effect of each of the nine cleaning strategies in each of the five patients.

**Results:**

A statistically significant difference in the efficacy of the cleaning strategies was detected. Specifically, rinsing with water alone was significantly less efficacious, and a combination of cationic detergent solution and ultrasonication was significantly more efficacious than the other methods (*p* < 0.05).

**Conclusions:**

Of the nine cleaning strategies examined, only that involving 5 min of ultrasonication at 42 k Hz combined with a 0.3% germicidal cationic detergent was observed to be statistically effective at removing the bacterial biofilm from the surface of F22 aligners.

## Background

A recent survey by YouGov estimated that 45% of adults are unsatisfied with their smile and that 20% would like to undergo orthodontic treatment to improve their tooth alignment and appearance (www.bos.org.uk/news/NOWYouGovSurvey). Hence, the orthodontics industry has paid great attention to the aesthetics of orthodontic appliances, seeking to make them as invisible as possible [1]. In this context, in 1997, Zia Chishti and Kelsey Wirth developed a barely noticeable aligner system, which they called Invisalign [2]. This system involves the use of a series of customised aligners made of transparent plastic; if worn for a minimum of 20 h per day and replaced every 2 weeks, this system can achieve dental movements of approximately 0.25–0.33 mm per tooth, or group of teeth, per aligner. Dental movement and malocclusion correction stages are planned using virtual planning software 3D (ClinCheck), based on CAD-CAM technology, which is also used to view the final result before treatment is begun [3].

There are several other advantages to clear aligner systems, including aesthetics, comfort, chairside time reduction, and the fact that they can be removed for meals and oral hygiene procedures [4]. However, like any orthodontic device, aligners do contribute to a worsening of oral health due to the accumulation of biofilm. Indeed, biofilm deposition on aligner surfaces has been clearly documented and its impact on oral health requires careful investigation [5, 6]. That being said, there have thus far been very few studies to analyse the impact of clear orthodontic aligners on the oral ecosystem, and their consequent influence on caries formation and decalcification. Nevertheless, it has been shown that adults in active treatment with clear aligners have better periodontal health than those undergoing fixed multibracket appliance treatment, who show worse plaque index, bleeding on probing and periodontal pocketing [7]. This is likely due to the fact that clear aligners can be removed and, therefore, cleaned more effectively to remove dental plaque—a complex community of microorganisms existing on the surface of the teeth in a polymeric matrix of bacterial origin. This biofilm is the cause of many oral diseases, and it has been estimated that roughly 60% of human infections can be ascribed to microbial biofilms [8].

Recent research has shown that a minimal dose of chlorhexidine (0.06%) has no beneficial effect on the oral health of aligner wearers [9], who must keep their appliances in place for 22 h per day in order to ensure good orthodontic outcomes (though it should be mentioned that many patients also keep their aligners on while eating and drinking). While they are being worn, aligners accumulate plaque and a bacterial biofilm forms around the teeth [10]. Aligners also limit the buffering, detergent and remineralising effects of saliva.

The biofilm on the enamel is not disturbed by the mechanical action of the lips, cheeks and tongue if an aligner is in place. In fact, one of the main characteristics of the biofilm is the cellular adhesion that occurs between microorganisms and non-exfoliative surfaces. The structure of the biofilm changes according to the bacterial species it is composed of [11–14], but the way in which it is organised protects against the action of chemical and pharmaceutical agents. Indeed, all infections closely linked to the development of the biofilm are highly resistant to non-invasive treatments (i.e. pharmacological) [12, 15, 16].

However, it has been demonstrated that oral bacteria can be destroyed by ultrasonication, via a mechanical phenomenon known as cavitation (bubble formation) [17]. Hence, in a recent study, published by the *Journal of Clinical Orthodontics* in 2013, Moshiri et al. [10] included this method of cleaning in a list of recommendations they drew up for orthodontists. They also included it in the suggested home hygiene protocol to be given to patients in order to ensure optimal outcomes in aligner therapy. In particular, they advised that patients should refrain from eating with their aligners in, remove any white deposits from their aligners, brush their teeth with a soft brush for two minutes, use dental floss and rinse with fluoride mouthwash in the evening, and always put clean aligners into a clean mouth. For aligner cleaning, they advised the use of either an ultrasonic bath or the Invisalign Cleaning System detergent. However, they cited no specific information regarding the efficacy of these methods.

Hence, in order to provide more information on the topic, we set out to conduct a comparative SEM (scanning electron microscopy) study of various appliance cleaning strategies in real-world patients being treated using F22 aligners [18, 19].

## Methods

Five patients were scheduled for orthodontic treatment via clear aligners. Silicon impressions were taken of their upper and lower teeth using the dual-impression method. Class 4 plaster (Ortotypo, Lascod®) was used to make the casts, which were then scanned in the lab using an extraoral scanner (SMART, Open Technologies), to obtain STL files and 3D renderings of the patients’ dentition. These were then converted into resin models using a laser printer (EnvisionTEC ULTRA 3SP), which were in turn used as moulds for a series of nine F22 aligners [18, 19], formed out of thermoplastic polyurethane (TPU), after isolating them with Isofolan sheets.

Each aligner in the series was worn by the patient for 14 days, being removed only at meals and during oral hygiene procedures. Patients were instructed to clean the nine aligners using the following strategies, respectively:

1. Rinsing with water
2. Immersion in water in a sonic bath
3. Immersion in water in an ultrasonic bath
4. Immersion in water and anionic detergent
5. Immersion in water and anionic detergent in a sonic bath
6. Immersion in water and anionic detergent in an ultrasonic bath
7. Immersion in water and cationic detergent
8. Immersion in water and cationic detergent in a sonic bath
9. Immersion in water and cationic detergent in an ultrasonic bath

The sonic device used (TCS Fresh®) for aligners 2, 5 and 8 in the series features a bath in which to immerse the aligner and vibrates at a frequency 5800 Hz, while the ultrasonic device (iSonic F3900®) used for aligners 3, 6 and 9, though very similar in structure, vibrates at a frequency of 42,000 Hz. According to the manufacturer’s instructions, the anionic detergent used (TCS Fresh®) for aligners 4, 5 and 6 should not be used directly in the mouth or for more than 7 days. It is sold in powder form in 1.75-g packets, each to be dissolved in 100 ml of water. Similarly, direct contact with the cationic detergent (benzalkonium chloride, Caelo®) used with aligners 7, 8 and 9, sold in a 0.3% aqueous solution, should also be avoided, as it is toxic to the skin and upon ingestion, and can irritate the skin and eyes. Before replacing in the mouth, therefore, patients were instructed to thoroughly rinse their aligners under running water to remove any trace of detergent. Aligner cleaning times for each strategy were standardised at 5 min, and each disinfection procedure was performed by the patient at home twice a day.

**Fig. 1:**
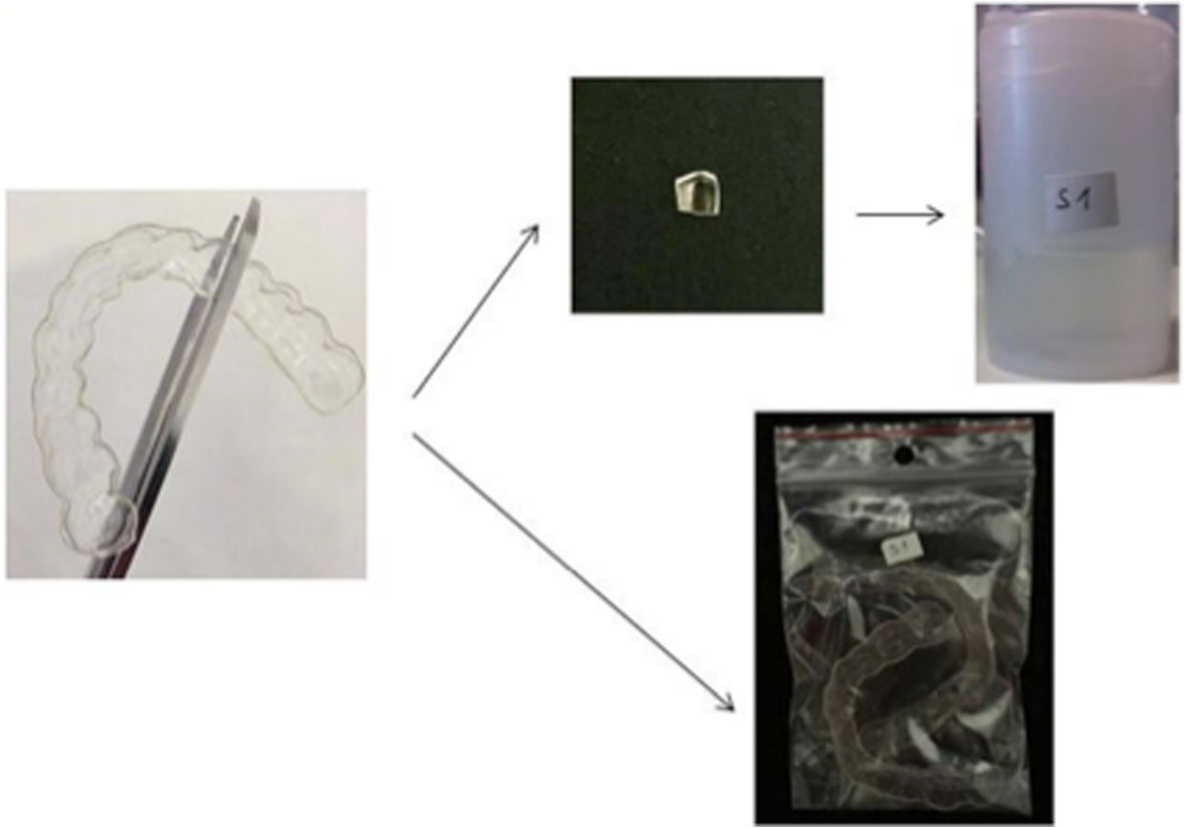
Processing a used aligner before sending to the lab

After being worn for 14 days, the aligners were returned to the orthodontist for analysis. Specifically, a 6 × 6-mm section of each aligner was cut from the vestibular surface of the upper right first premolar area (the transition zone between anterior and posterior sectors). Each section was immediately conserved in glutaraldehyde, and the aligner sealed in a plastic bag; both the container of glutaraldehyde were labelled with the same ID (Fig. 1). The preserved aligner sections were dried and gilded at the University of Ferrara Microscopy Lab and then observed under SEM. The scanning electron microscope was focused on the centre of each sample, which was enlarged 10 thousand times. The resulting SEM images were saved in JPEG format on an 8-bit colour-depth grey scale. A histogram of the grey-scale attributes was created for each SEM image to provide a graphical representation of the number of pixels in the image for each grey on the scale.

The dataset composed of the 45 observations, representing the measures on the grey scale (Value variable) of the nine different cleaning methods (one per aligner) used by the five patients, was then subjected to statistical analysis. Repeated-measures ANOVA was used to determine whether the Value variable was influenced by the different aligner/cleaning strategy combinations. The software R nlme [20] was used for ANOVA modelling, specifying “cleaning strategy” as a “within subject” variable, and the patient as a random factor. In order to identify statistically significant differences between aligner and cleaning strategy combinations, a post hoc analysis was performed using Multcomp software [21]. This analysis showed that the differences between the effects of distinct cleaning strategy pairs were statistically equal to zero.

## Results

**Fig. 2:**
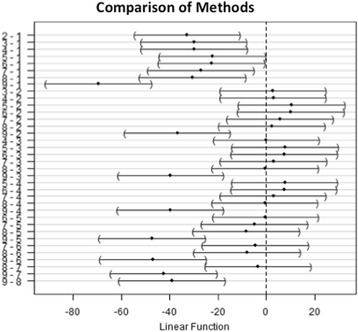
Post hoc analysis of cleaning strategy pairs

The SEM image analysis (Table 1) and statistical analysis summarising the Value measurements per aligner/cleaning strategy (Table 2) show that the cleaning strategy variable does have a statistically significant effect on the Value (*p* value zero), i.e. the “cleanliness” of the aligner. However, as shown in Table 3 and Fig. 2, only the first and ninth cleaning strategies (rinsing with water and immersion in water and cationic detergent in an ultrasonic bath, respectively) were significantly different from the others, the former being significantly less efficacious, and the latter being significantly more efficacious.

**Table 1:** The mean grey values of each image of aligners cleaned by each of the nine cleaning strategies in each of the five patients, showing the mean calculated for each cleaning method

	Method 1	Method 2	Method 3	Method 4	Method 5	Method 6	Method 7	Method 8	Method 9
PT 1	143.618	126.714	111.379	112.007	109.033	124.756	132.793	117.481	81.492
PT 2	154.730	115.470	111.380	109.226	109.219	132.481	131.457	114.486	83.033
PT 3	140.032	107.871	112.168	140.546	133.622	107.512	125.045	129.604	84.454
PT 4	163.922	114.175	131.064	108.467	145.483	138.692	117.787	119.598	80.219
PT 5	147.972	122.249	134.454	130.679	141.776	134.050	108.033	116.978	73.534
MEAN	150.055	117.296	120.089	120.185	127.827	127.498	123.023	119.629	80.546

**Table 2:** Summarises the values for each cleaning strategy: number of observations, mean grey value and standard deviation

Method	Length	Mean	SD
1	5	150.1	9.492
2	5	117.3	7.334
3	5	120.1	11.63
4	5	120.2	14.57
5	5	127.8	17.6
6	5	127.5	12.25
7	5	123	10.28
8	5	119.6	5.865
9	5	80.55	4.232

**Table 3:** A *p* value of zero indicates that the ANOVA test used is significant and refutes the hypothesis that all methods tested are similar

	numDF	denDF	*F* value	*p* value
(Intercept)	1	32	5317	0
Method	8	32	13.13	0.00000003592

## Discussion

All aligner cleaning strategies did reduce the bacterial biofilm on the surface of our aligner samples, with the exception of rinsing under tap-water alone. However, our data also show that the most efficacious strategy tested was cationic detergent combined with ultrasonication. Our comparative results also appear to confirm the literature reports that the only way to kill bacteria via low-intensity ultrasound is by combining it with a bactericidal agent [22, 23]. Indeed, we clearly show that a cationic detergent alone is not sufficient to eliminate the bacterial biofilm from the surface of aligners, despite its bactericidal properties, which have been demonstrated in other studies [24, 25]. Similarly, 5 min of 42,000 Hz ultrasound alone was not sufficient to remove the biofilm [26], and only a combination of the two methods proved useful in this regard according to our statistical analysis.

Indeed, even a brief glance at the SEM images for each of the aligners reveals evident bacterial colonisation of each, with the exception of those cleaned by the ninth strategy, which combined an ultrasonic bath with a cationic bactericide. These compare very favourably not only with those rinsed with water alone, which show visible layers of abundant bacteria organised into a biofilm, but also those cleaned using the other strategies (2–8), which show variable levels of bacterial proliferation.

**Fig. 3:**
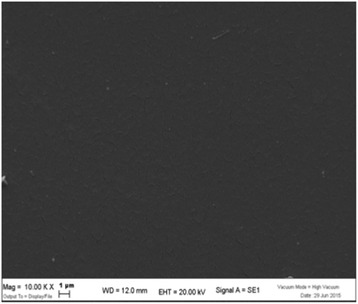
Unused aligner

**Fig. 4:**
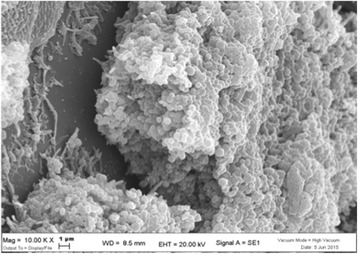
Aligner cleaned by rinsing with water alone (method 1)

As the SEM image of the unused aligner (Fig. 3) is much darker than that cleaned by the first method (Fig. 4)—rinsing with water alone—we decided to numerically quantify the visible biofilm on the aligner sections using grey-scale analysis, taking a mean of the levels of grey in the images of the aligners cleaned by each method. The cleaner aligners are darker in colour and show grey-scale levels closer to that of the unused aligner, i.e. 61.924, which appears totally dark. Bacterial proliferation on the surface of the aligner, on the other hand, makes them appear lighter in colour, and the multi-layer biofilm causes the mean level of grey to differ considerably from that of the clean TPU of the unused aligner. By statistically analysing this data, we were able to confirm that there was indeed a statistically significant relationship between the SEM grey-scale levels and the cleanliness of the aligner sections.

**Fig. 5:**
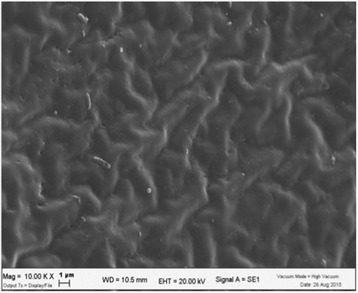
Aligner cleaned by method 9 shows clear signs of water absorption and ultrasonic cavitation

Careful SEM analysis of the aligner sections cleaned via the ninth method (Fig. 5), i.e. ultrasonic bath and cationic detergent, reveal interesting points regarding their surface characteristics. The first is that the TPU material itself bears clear striations, an indication of its hydrophilic status [25, 26], and its absorption of water. The second visible feature is related to the method of cleaning used; the surface of the aligner bears signs of pitting. This is likely an effect of ultrasonic cavitation and is visible thanks to the lack of bacterial proliferation. It demonstrates that the mechanical turbulence generated by the acoustic waves, the continual formation and rupture of tiny bubbles, releases sufficient energy to damage not only bacterial cell walls [27] but also the surface of thermoplastic polyurethane.

In order to directly compare these findings with those in the literature in any meaningful way, we would need to rely on studies conducted using the same methods for analysing the aligner surfaces and counting the bacterial colonies. However, there is great variability in the literature in terms of these factors, and in any case, our design prevented us from providing precise bacterial counts for comparison. Nevertheless, our findings generally confirm those of Torlak and Sert [28] and Mermillod-Blondin et al. [29] that the association between a germicidal detergent and ultrasonication is able to reduce the bacterial load on the surfaces analysed.

## Conclusions

Of the nine cleaning strategies examined, only that involving 5 min of ultrasound treatment at 42 kHz combined with a 0.3% solution of the germicidal cationic detergent benzalkonium chloride was statistically observed to be effective at removing the bacterial biofilm from the surface of used F22 TPU aligners [18, 19] (*p* < 0.05).
